# Exploring microbiome engineering as a strategy for improved thermal tolerance in *Exaiptasia diaphana*

**DOI:** 10.1111/jam.15465

**Published:** 2022-02-19

**Authors:** Ashley M. Dungan, Leon M. Hartman, Linda L. Blackall, Madeleine J. H. van Oppen

**Affiliations:** ^1^ School of BioSciences The University of Melbourne Melbourne Victoria Australia; ^2^ Swinburne University of Technology Hawthorn Victoria Australia; ^3^ Australian Institute of Marine Science Townsville Australia

**Keywords:** assisted evolution, coral bleaching, *Exaiptasia diaphana*, microbiome engineering, microbiome manipulation, ROS

## Abstract

**Aims:**

Fourteen percent of all living coral, equivalent to more than all the coral on the Great Barrier Reef, has died in the past decade as a result of climate change‐driven bleaching. Inspired by the ‘oxidative stress theory of coral bleaching’, we investigated whether a bacterial consortium designed to scavenge free radicals could integrate into the host microbiome and improve thermal tolerance of the coral model, *Exaiptasia diaphana*.

**Methods and Results:**

*E.* *diaphana* anemones were inoculated with a consortium of high free radical scavenging (FRS) bacteria, a consortium of congeneric low FRS bacteria, or sterile seawater as a control, then exposed to elevated temperature. Increases in the relative abundance of *Labrenzia* during the first 2 weeks following the last inoculation provided evidence for temporary inoculum integration into the *E.* *diaphana* microbiome. Initial uptake of other consortium members was inconsistent, and these bacteria did not persist either in *E. diaphana*’s microbiome over time. Given their non‐integration into the host microbiome, the ability of the FRS consortium to mitigate thermal stress could not be assessed. Importantly, there were no physiological impacts (negative or positive) of the bacterial inoculations on the holobiont.

**Conclusions:**

The introduced bacteria were not maintained in the anemone microbiome over time, thus, their protective effect is unknown. Achieving long‐term integration of bacteria into cnidarian microbiomes remains a research priority.

**Significance and Impact of the Study:**

Microbiome engineering strategies to mitigate coral bleaching may assist coral reefs in their persistence until climate change has been curbed. This study provides insights that will inform microbiome manipulation approaches in coral bleaching mitigation research.

## INTRODUCTION

The persistence of coral reefs is threatened by elevated sea surface temperature (SST) and associated summer heat waves that are the result of climate change (Hughes et al. 2017). As the dominant primary producers and reef builders, scleractinian corals are critical components of coral reefs. Corals live in symbiotic associations with other organisms, including bacteria, protists, fungi, archaea and viruses (Blackall et al. 2015; Ricci et al. 2019; Ainsworth et al. 2020). These microbial associates contribute to the health of this complex host–microbe association, or holobiont (Rohwer et al. 2002). Crucial members of the coral holobiont are the dinoflagellate photosymbionts of the family Symbiodiniaceae. In this mutually beneficial relationship, corals provide a safe haven and inorganic nutrients, while the Symbiodiniaceae meet most of the coral’s energy needs through the transfer of photosynthate in a nutrient‐poor seawater environment (Muscatine et al. 1981; Yellowlees et al. 2008; Tremblay et al. 2014). The relationship between the coral host and Symbiodiniaceae can break down during periods of stress, resulting in the loss of the symbionts from the coral tissues, a process called coral bleaching.

There are several hypotheses for the mechanisms that drive bleaching (Weis 2008; Cunning and Baker 2013; Wiedenmann et al. 2013; Wooldridge 2013), with a common theme being the overproduction of reactive oxygen species (ROS) by the algal symbiont and their toxic accumulation. Elevated SST inhibits Symbiodiniaceae photosynthesis via damage to photosystem II (PSII) (Warner et al. 1999; Tchernov et al. 2004). This impairment leads to increased levels of ROS, which, once generated, can trigger the oxidation of essential photosynthetic molecules (Wang et al. 2011; Mathur et al. 2014), thylakoid membranes (Tchernov et al. 2004; Roberty et al. 2016), and enzymes of the Calvin‐Benson cycle (Asada and Takahashi 1987; Lesser and Farrell 2004), thereby interfering with the supply of fixed carbon to the holobiont (Lesser 2004). Increased abundance of ROS in the chloroplast also reduces the biosynthesis of chlorophyll (Takahashi et al. 2008; Roberty et al. 2016).

In the ‘oxidative stress theory of coral bleaching’ (Downs et al. 2002), ROS produced by the Symbiodiniaceae are thought to diffuse into host cells and activate signalling cascades resulting in the loss of Symbiodiniaceae from the coral host (Smith et al. 2005; Weis 2008; Davy et al. 2012; Suggett and Smith 2020). If separation of coral and Symbiodiniaceae occurs, it may be fatal for the coral unless the symbiosis is re‐established. Thus, with average SST predicted to rise further and associated summer heat waves increasing in frequency, severity and duration (Aral and Guan 2016), finding effective coral bleaching mitigation methods has become crucial.

Many innovative approaches have been proposed to mitigate bleaching and enhance coral survival during thermal stress, including selective breeding of thermally tolerant coral genotypes and the creation of interspecific hybrids (van Oppen et al. 2017; Chan et al. 2018; Quigley et al. 2020), directed evolution of Symbiodiniaceae (Chakravarti et al. 2017; Buerger et al. 2020), and inoculation with bacteria to engineer the coral microbiome (van Oppen et al. 2015; Damjanovic et al. 2017; Peixoto et al. 2017; Blackall et al. 2020; Peixoto et al. 2021). Directly targeting ROS, however, has rarely been investigated, despite evidence that exposure to exogenous antioxidants can reduce Symbiodiniaceae loss (Lesser 1997), increase survival (Nesa and Hidaka 2009), reduce respiration rates (Lesser 1997), and decrease DNA damage (Nesa and Hidaka 2008; Majerová and Drury 2021) in scleractinian corals exposed to thermal stress. The cumulative results from these studies suggest that increased biologically available antioxidants could potentially mitigate bleaching and prolong the life of corals during climate change induced summer heat waves. Nevertheless, applying antioxidants directly onto corals may not be practical due to their short lifespan when exposed to seawater (King et al. 2016) and ultraviolet radiation (Compton et al. 2019), or desirable due to their potential impact on non‐target organisms. Instead, increasing natural antioxidant generation within the coral holobiont may be more effective and feasible.

Microbiome manipulation has been suggested as a method for increasing natural antioxidant generation in the cnidarian holobiont (van Oppen et al. 2015; Epstein et al. 2019). Exposure to beneficial bacteria has reduced the impact of pathogens (Alagely et al. 2011) or environmental stressors such as oil pollution (dos Santos et al. 2015) on cnidarians. Inoculation of coral with native bacteria (Doering et al. 2021), including catalase‐positive isolates, has also improved bleaching tolerance in corals exposed to elevated temperature (Rosado et al. 2018; Santoro et al. 2021). Although the results of the latter studies were promising, the reason for improved bleaching tolerance was unclear due to the selected bacteria’s broad range of traits. Furthermore, the stressed corals may have been supported indirectly through heterotrophic feeding on the introduced bacteria (Houlbrèque et al. 2004; Meunier et al. 2019) as the control corals in both cases were starved. Consequently, the influence of antioxidant‐producing bacteria on cnidarian bleaching requires further investigation.

A major challenge with bacterial inoculation therapies is transient colonization (Hai 2015), with continuous addition of bacteria required for long‐term maintenance. Given the scale of coral reefs, repeated dosing with bacteria is not a feasible long‐term strategy to mitigate coral bleaching. However, if the bacteria can form a stable association with the host, the benefits of inoculation may persist overtime.

To assess the ability of introduced bacteria to form a stable presence in the cnidarian microbiome and evaluate their influence on host health and bleaching, we inoculated the sea anemone *Exaiptasia diaphana* with a bacterial consortium consisting of host‐derived free radical scavenging (FRS) bacteria (Dungan et al. 2021a) prior to thermal stress. Metabarcoding of bacterial 16S rRNA genes was used to track the incorporation of the bacteria into the *E*. *diaphana* microbiome and changes in bacterial community structure across time and treatments. The holobiont’s bleaching response to thermal stress was assessed by measuring Symbiodiniaceae photosynthetic performance and cell densities, while net ROS was quantified in host tissues with a fluorescent reagent. The influence of host genotype and Symbiodiniaceae strain was also explored by comparing the responses of three *E. diaphana* genotypes and tracking Symbiodiniaceae community composition.

## METHODS

### Selection of free radical scavenging bacteria

Six *E. diaphana*‐sourced bacterial isolates with high FRS ability were selected based on their ability to neutralize a stable free radical for inclusion in a bacterial consortium, with six conspecific/congeneric strains with low FRS ability selected for inclusion as a negative control (Table 1). Identification of the bacterial candidates and their phenotypic ability to scavenge free radicals has been previously described (Dungan et al. 2021a), but see supplementary file S1 for an overview.

**TABLE 1:** High FRS and low FRS bacteria and their 16S rRNA gene GenBank accession numbers. ‘+’ and ‘−’ indicate high and low FRS strains, respectively, for the congeneric *Micrococcus* strains

Family	Genus	Species	High FRSStrainID/NCBI accession number	Low FRSStrainID/NCBI accession number
Alteromonadaceae	*Alteromonas*	*oceani*	MMSF01163/MN540711	MMSF00404/MN540719
Alteromonadaceae	*Alteromonas*	*macleodii*	MMSF00958/MN540717	MMSF00257/MN540713
Alteromonadaceae	*Marinobacter*	*salsuginis*	MMSF01190/MN540716	MMSF00964/MN540714
Flavobacteriaceae	*Winogradskyella*	*poriferorum*	MMSF00046/MN540715	MMSF00910/MN540718
Micrococcaceae	*Micrococcus*	*luteus* (+), *yunnanensis* (−)	MMSF00068/MN540712	MMSF00107/MN540722
Rhodobacteraceae	*Labrenzia*	*aggregata*	MMSF00132/MN540721	MMSF00249/MN540720

To prepare the high and low‐FRS consortia, bacterial isolates were first grown from cryopreserved cells on Reasoner’s 2A (R2A, Oxoid) solid media supplemented with Red Sea Salt™ seawater (R11065, Red Sea), hereafter ‘RSS’, to 34 parts per thousand (ppt) salinity to confirm purity. Individual isolates were selected and grown to log phase in 50 ml of modified R2A broth (mR2A; Table S1). The FRS phenotype of each high and low FRS bacterial strain was similar when incubated at 26°C or 37°C **(** Table S2 **;** Dungan et al. 2021a **)**. Since the bacteria grew more rapidly at 37°C, cultures were incubated at 37°C for 48 h at 150 rpm in an orbital incubator (OM11, Ratek). Uninoculated broth was also incubated to confirm medium sterility. Three replicate cultures were grown per isolate to ensure sufficient biomass was available for the inoculations. After 48 h, the culture and medium blanks were centrifuged at 3000 × *g* at 4°C for 30 min to pellet the bacteria. The pelleted cells were washed three times by resuspending in 10 ml 0.2 μm‐filtered RSS (fRSS) reconstituted at ~34 ppt salinity (kept at 4°C), and centrifuged at 3000 × *g* at 4°C for 15 min. After the final centrifugation, the pellet was resuspended in 5 ml fRSS and the three replicate cultures per isolate were combined. Optical density measurements at 600 nm (OD_600_; CLARIOstar PLUS plate‐reader, BMG Labtech) were taken of the pooled triplicates at a 1:10 dilution in fRSS, the total number of cells per isolate was calculated based on *Escherichia coli* values (Volkmer and Heinemann 2011), and the pools were centrifuged and resuspended to a density of 10^9^ cells ml^−1^. Each bacterial suspension was pooled into the two different bacterial treatments. As OD_600_ readings are influenced by cell size, shape, and colour, and do not discriminate between living and dead cells, the density of viable cells for each isolate was confirmed by counting colony‐forming units (CFUs). Three replicate plates of R2A were spread inoculated with 50 μl of four serial dilutions (10^−5^ to 10^−8^) of each isolate. The plates were then incubated at 26°C for 7 days before CFUs were counted.

### Experimental set‐up

Anemones from cultures of three Great Barrier Reef (GBR)‐sourced *E. diaphana* genotypes, AIMS2, AIMS3 and AIMS4, were randomly selected from The University of Melbourne culture collection (*n* = 450 per genotype) (Dungan et al. 2020). Anemones from each genotype were equally distributed among 18 × 300 ml lidded glass culture jars (total jars = 54) and the jars were evenly split between two experimental incubators (Hi‐Point 740FHC, Thermo Fisher) fitted with red, white and infrared light‐emitting diode (LED) lights. All anemones were maintained in RSS at ~34 ppt salinity and fed ad libitum twice weekly with freshly hatched *Artemia salina* (Salt Creek, Premium GSL). Jars were cleaned periodically by loosening algal debris with seawater pressure applied through sterile plastic pipettes followed by full RSS changes. All anemones were transferred to clean jars twice during the study period to combat algal growth. Seawater temperatures were monitored using submersible data loggers (Hobo UA‐001‐08, OneTemp). The anemones were acclimated to the experimental incubators for 3 weeks. Initial light levels were 12 μmol photons m^−2^ s^−1^ to match the stock culture conditions, then gradually increased to 28 μmol photons m^−2^ s^−1^ over 72 h on a 12 h:12 h light: dark cycle beginning at 6 a.m. Our experimental light intensity corresponds with previous studies evaluating bleaching via thermal stress on *E*. *diaphana* (Tolleter et al. 2013; Bieri et al. 2016).

### Treatment schedule

**FIGURE 1:**
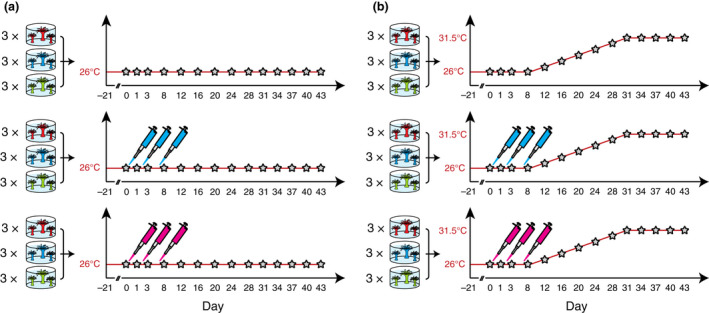
Inoculation and sampling schedule for each inoculum‐temperature combination. Temperature conditions: (a) ambient (26°C), and (b) elevated (26–31.5°C). High (blue pipettors) and low (pink pipettors) FRS inoculations were performed on days 0, 2 and 7. Stars indicate sample collection timepoints. At each sample collection timepoint, one anemone from each of three replicate culture jars for three *E. diaphana* genotypes (AIMS2, AIMS3, AIMS4) was collected. Total anemone samples, *n* = 756

The 54 experimental glass culture jars each contained 24 anemones and were split between 18 treatments (three bacteria inocula × two temperatures × three anemone genotypes), with three replicate jars per treatment. The experiment began after the 3‐week acclimation period at a timepoint designated Day 0 (Figure 1). On Day 0 at 10 h into the light cycle, treated anemones were inoculated with the high or low FRS bacterial consortia. A volume of 1 ml of the respective consortium at 10^9^ bacterial cells ml^−1^ (described above) was pipetted into glass jars containing 300 ml RSS and anemones. The final density was 10^6^ bacterial cells ml^−1^ of each species, in line with previous inoculation studies (Rosado et al. 2018; Damjanovic et al. 2019b) and far exceeds the described bacterial carrying capacity of *E*. *diaphana* of 10^3^–10^5^ bacteria anemone^−1^ (Costa et al. 2021; Dungan et al. 2021a). No‐inoculum control anemones were not inoculated with bacteria. *A. salina* nauplii (approx. 1000) were administered during each treatment to induce feeding and encourage ingestion of the consortia. Anemones were maintained in seawater with inoculum for ~16 h after which each jar was rinsed 3x with RSS. The treated anemones were inoculated again on Days 2 and 7 with fresh bacteria and *A. salina*. All anemones were exposed for 43 days to ambient temperature (26°C), or at temperature increasing from ambient to 31.5°C between Day 8 and Day 31 (0.25°C/day), then held at 31.5°C. The temperature ramp rate was designed to approximate GBR summer heatwave conditions (Figure S1, AIMS 2019). The temperature designation of the incubators was swapped fortnightly and the anemones were randomly rearranged each week to remove incubator and jar position as confounding factors.

### Symbiodiniaceae photosynthetic efficiency measurement

Photosynthetic efficiency, measured as the maximum quantum yield (*F*_*v*_/*F*_*m*_) of PSII of Symbiodiniaceae, is a commonly used proxy for general holobiont health as lowered *F*_*v*_/*F*_*m*_ indicates PSII damage resulting from thermal stress or high irradiance (Ralph et al. 2016). *F*_*v*_/*F*_*m*_ was measured on the intracellular Symbiodiniaceae by pulse‐amplitude‐modulated (PAM) fluorometry with an imaging‐PAM system (IMAG‐MAX/L, Walz Heinz, Germany). On sampling days, measurements were taken from the bodies and proximal tentacles of five anemones per jar 4 h after the light cycle started and after 30 min dark adaptation. PAM settings: saturating pulse intensity 8, measuring light intensity 2 (frequency 1), actinic light intensity 3, damping 2 and gain 2.

### Anemone tissue processing

On sampling days, one anemone per jar was sacrificed for Symbiodiniaceae cell density, ROS, and protein quantification, and DNA extraction for bacterial community analysis. Each anemone was individually homogenized in a sterile glass homogenizer in 1 ml of fRSS and an aliquot of 250 μl was flash frozen in liquid nitrogen for DNA extraction. The remaining homogenate was centrifuged at 5000 × *g* for 5 min at 4°C to pellet the Symbiodiniaceae, and 50 μl of the supernatant was stored at −20°C for anemone (host) protein measurement, while 594 μl of supernatant was taken for ROS quantification. Any remaining supernatant was discarded.

### Symbiodiniaceae cell density measurement

Pelleted Symbiodiniaceae were washed twice with 750 μl fRSS and centrifuged at 5000 × *g* for 5 min at 4°C, then resuspended in 750 μl fRSS. Triplicate cell counts (cells ml^−1^) were performed on 10 μl of sample within 48 h of sample collection with an automated cell counter (Countess II FL, Life Technologies). Cell counts were normalized to host protein (mg ml^−1^) determined by the Bradford assay (Bradford 1976). Each protein sample was assayed in triplicate and measured at 595 nm (EnSpire MLD2300 plate‐reader, Perkin Elmer) against bovine serum albumin standards (500–0207, Bio‐Rad).

### ROS assay

Anemone net ROS levels, defined as the difference between ROS produced and ROS scavenged by enzymatic and non‐enzymatic antioxidants, were quantified with CellROX® Orange (C10443, Thermo Fisher, Australia) (Levin et al. 2016; Chakravarti et al. 2017; Gegner et al. 2019; Buerger et al. 2020). For each sample, 594 μl of host tissue supernatant, collected as above, was vortexed briefly with 6 μl CellROX® Orange (final concentration 5 μM) and incubated in darkness at 37°C for 30 min. Three 200 μl technical replicates of each sample were transferred into a 96‐well black, clear‐bottom culture plate (Nunc 165,305, Thermo Fisher). Fluorescence readings at excitation/emission of 545/565 nm (EnSpire MLD2300, Perkin Elmer) were then taken and the average reading per sample was normalized to host protein (mg ml^−1^) to give net ROS values in arbitrary fluorescent units.

### Metabarcoding sample preparation

DNA was extracted from the anemone samples (Wilson et al. 2002; Hartman et al. 2019). Sample‐free negative controls (*n* = 12) were also processed to identify contaminants introduced during DNA extractions. Extracted DNA and negative controls were amplified by PCR in triplicate using bacterial primers with overhang adapters (underlined) designed at the Walter and Eliza Hall Institute (WEHI), Melbourne, Australia, targeting the V5–V6 regions of the 16S rRNA genes: 784F [5′ GTGACCTAT GAACTCAGGAGTCAGGATTAGATACCCTGGTA 3′]; 1061R [5′ CTGAGACTTGC ACATCGCAGCCRRCACGAG CTGACGAC 3′] (Andersson et al. 2008). Template‐free negative controls (*n* = 6) were included to identify contaminants introduced during PCR.

Anemone samples from Days 0 and 43 were also amplified with primers to characterize the Symbiodiniaceae population by targeting the internal transcribed spacer 2 (ITS2) region of the rRNA gene using primers Sym_Var_5.8S2 [5’ GTGACCTATGAA CTCAGGAGTCGAATTGCAGAA CTCCGTGAACC 3′] (Hume et al. 2015); Sym_Var_Rev [5’ CTGAGACTTGC ACATCGCAGCCGGGTTCWCTT GTYTGACTTCATGC 3′] (Hume et al. 2013). Each PCR contained 7.5 μl MyTaq HS Mix polymerase (Bioline), 1 μl of DNA template, 0.3 μmol L^−1^ of each primer, and nuclease‐free water up to 15 μl. PCR conditions were: 1 cycle × 95°C for 3 min; 18 cycles × 95°C for 15 s, 55°C for 30 s, and 72°C for 30 s; 1 cycle × 72°C for 7 min; hold at 4°C. The triplicate PCR products from each sample were then pooled.

DNA sequencing libraries were prepared according to Aubrey et al. (2015). Briefly, 20 μl of each PCR product pool was purified by size‐selection using NucleoMag® NGS Clean‐up and Size Select magnetic beads (Macherey‐Nagel, Scientifix). The purified DNA was resuspended in 40 μl nuclease‐free water. Indexing PCRs were created by combining 10 μl of each DNA suspension with 10 μl 2× Taq master mix (M0270S, New England Biolabs) and 0.25 μmol L^−1^ of forward and reverse indexing primers. PCR conditions were: 1 cycle × 95°C for 3 min; 24 cycles × 95°C for 15 s, 60°C for 30 s, and 72°C for 30 s; 1 cycle × 72°C for 7 min; hold 4°C. Fifty random samples were checked for product size and quantity (2200 TapeStation, Agilent Technologies). Sequencing libraries were created by pooling 5 μl from each reaction by plate and performing a final bead clean‐up on 50 μl. Each library was checked for quality and quantity to guide pool normalization (2200 TapeStation), then sequenced across three Illumina MiSeq runs using v3 (2 × 300 bp) reagents at WEHI.

### Metabarcoding data processing

Raw 16S rRNA gene sequences were imported into QIIME2 v2019.10.0 (Bolyen et al. 2019) and demultiplexed on a per‐sequencing‐run basis. Primers were removed with cutadapt v2.6 (Martin 2011). Filtering, denoising, and chimera checking was performed using DADA2 (Callahan et al. 2016) in the QIIME2 environment to correct sequencing errors, remove low‐quality bases (mean Qscore <30), and generate bacterial amplicon sequence variants (ASVs). Data from each sequencing run were then merged, and taxonomy for each ASV was assigned against a SILVA database (version 132) trained with a naïve Bayes classifier against the same V5‐V6 region targeted for sequencing (Bokulich et al. 2018).

### Identification of Symbiodiniaceae genetic diversity

Raw sequences for each sample (*n* = 108) were processed using the SymPortal analytical framework (symportal.org) (Hume et al. 2019). Sequence information was submitted to the SymPortal remote database and underwent quality control including the removal of artefact and non‐Symbiodiniaceae sequences. Within‐sample informative intragenomic sequences, referred to as ‘defining intragenomic variants’ (DIVs) were used to identify ITS2‐type profiles of putative Symbiodiniaceae taxa (Hume et al. 2019). ITS2 type profiles were designated by SymPortal based on the presence and abundance of the ITS2 sequences in our samples and within the SymPortal database.

### Data analysis

All data were analysed in R v3.6.2 (R Core Team 2020). Statistical tests were considered significant at *α* = 0.05. ASV, taxonomy, metadata and phylogenetic tree files were imported into R and combined into a phyloseq object (McMurdie and Holmes 2013). Potential contaminant ASVs were identified and removed from the dataset according to their abundance in the extraction (*n* = 12) and PCR (*n* = 6) negative controls relative to the anemone samples using the prevalence method in the R package decontam with *p* = 0.1 (Davis et al. 2018). Dark‐adapted *F*_*v*_/*F*_*m*_, Symbiodiniaceae cell density, and ROS measurements were plotted using the R package ggplot2 (Wickham 2016) with data separated by genotype. Data were analysed for overall differences using linear mixed effects (LME) models against the variables ‘inoculum’, ‘temperature’, and ‘time’, with ‘jar’ specified as a random effect, in the R package nlme (Pinheiro et al. 2020). Post hoc pairwise comparisons were performed using Tukey’s Honest Significant Difference test (Tukey 1949) in the R package emmeans (Searle et al. 1980) with Tukey’s adjustment for multiple comparisons. Shifts in the bacterial community compositions of each genotype were visualized in principal coordinate analysis (PCoA) ordinations based on Jaccard distance (Jaccard 1912). The influence of time, inoculum and temperature on changes in bacterial community composition within each genotype was investigated by comparison of generalized linear models (GLMs) using likelihood ratio tests across 999 iterations in the R package mvabund (Wang et al. 2012). For PCoAs and GLMs the ASV data were collapsed to genus for clarity and computational efficiency. Incorporation of bacteria into the anemone microbiomes was visualized in bubble plots to show changes in relative abundance at the ASV level for each anemone genotype, temperature, and inoculum over time. Apart from the *A. macleodii* strains, the ASV sequences of each high and low FRS pair were identical and therefore indistinguishable. Further tests with GLMs were performed, as above, to assess whether differences between the bacterial communities for each genotype‐temperature‐inocula combination were significant at selected time points.

## RESULTS

### FRS bacteria concentrations

The density of viable cells in the bacterial cultures for each inoculum was generally within one order of magnitude of the target of 10^9^ cells ml^−1^. Exceptions included the Day 7 *Winogradskyella* (low FRS) culture, which failed to produce colonies, Day 2 *Micrococcus* and *A. oceani*, and Day 7 *A. oceani* (high FRS) cultures, which had low viable cell numbers (Table 2).

**TABLE 2:** Cell density calculated from CFUs of high (left) and low (right) FRS bacteria cultured for their respective inocula with dosing on days 0, 2 and 7. Bacterial culture numbers <10^8^ cells ml^−1^ are in bold

High FRS strain	Dosing day	Concentration(cells ml^−1^)	Low FRS strain	Dosing day	Concentration(cells ml^−1^)
MMSF00046	**0**	**6.40 × 10** ^**7**^	MMSF00910	0	1.56 × 10^9^
*Winogradskyella*	2	2.34 × 10^8^	*Winogradskyella*	2	3.27 × 10^9^
	7	1.61 × 10^9^		**7**	**No growth**
MMSF00068	0	3.72 × 10^8^	MMSF00107	0	1.33 × 10^9^
*Micrococcus*	**2**	**3.80 × 10** ^**7**^	*Micrococcus*	2	2.37 × 10^9^
	7	1.29 × 10^9^		7	1.53 × 10^9^
MMSF00132	0	2.23 × 10^9^	MMSF00249	0	6.73 × 10^9^
*Labrenzia*	2	2.60 × 10^8^	*Labrenzia*	2	6.00 × 10^8^
	7	1.30 × 10^8^		7	7.73 × 10^8^
MMSF00958	0	2.19 × 10^8^	MMSF00257	0	4.80 × 10^9^
*Alteromonas macleodii*	2	1.43 × 10^8^	*Alteromonas macleodii*	2	2.53 × 10^9^
	7	4.37 × 10^8^		7	2.73 × 10^9^
MMSF01163	0	1.01 × 10^9^	MMSF00404	0	8.67 × 10^8^
*Alteromonas oceani*	**2**	**2.33 × 10** ^**7**^	*Alteromonas oceani*	2	3.87 × 10^8^
	**7**	**2.53 × 10** ^**7**^		7	4.07 × 10^9^
MMSF01190	0	1.87 × 10^9^	MMSF00964	0	9.27 × 10^8^
*Marinobacter*	2	2.45 × 10^8^	*Marinobacter*	2	4.67 × 10^8^
	7	3.39 × 10^9^		7	1.32 × 10^9^

### Bacteria metabarcoding data processing

Sequencing produced 16.8 M reads across 780 *E*. *diaphana* and control samples (minimum 2; mean 21,497, maximum 112,307 reads per sample). After merging, denoising and chimera filtering, 11.3 M reads remained (minimum 0, mean 14,506, maximum 91,465 reads per sample). Twenty‐one samples were identified with <100 reads per sample and were removed from the dataset. Decontam identified 209 putative contaminant ASVs, which constituted 1.36% relative abundance of the bacterial communities, and were removed (Table S3). After all filtering steps, there were 4555 ASVs across the remaining samples.

### Bacterial community shifts

**FIGURE 2:**
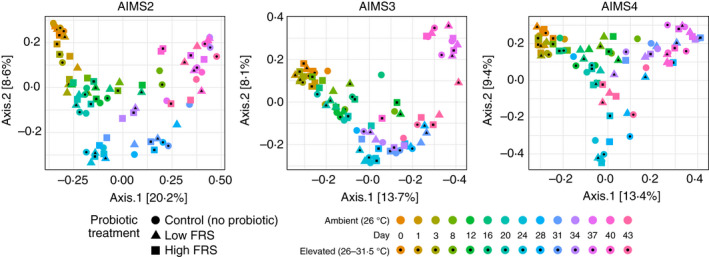
PCoA ordinations (Jaccard distance) for each anemone genotype depicting shifts in the anemone‐associated bacterial communities over time. For each datapoint, *n* = 3

A general temporal shift in the composition of the bacterial communities for each genotype was evident from the PCoA (Jaccard distance) plots (Figure 2, Figures S2–S4). There was no apparent clustering or separation of datapoints in the plots based on inoculum or temperature condition, and the primary driver of change in bacterial community composition was time.

### Incorporation of the FRS bacteria

**FIGURE 3:**
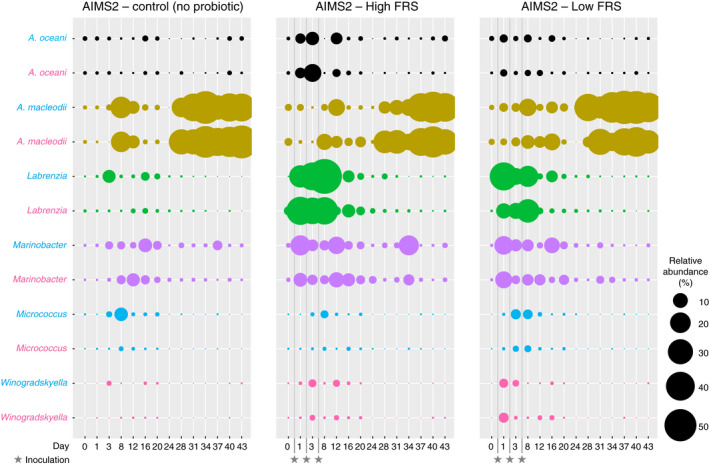
Relative abundance of genera of the inoculum bacteria in the AIMS2 anemones. Blue genus names = data for ambient temperature anemones (26°C). Pink genus names = data for elevated temperature anemones (26–31°C). Inoculations were performed at days 0, 2 and 7 and are indicated by stars. For each bubble, *n* = 3

Bubble plots illustrated shifts in the relative abundance of bacterial consortium members within the anemones, with results consistent between all anemone genotypes (Figure 3, Figures S5 and S6). The *A. oceani* ASV showed substantial increases after the first and second inoculations for the high FRS treatment, regardless of anemone genotype, and remained low in abundance in the no‐inoculum controls. In contrast, the relative abundance of *A. macleodii* ASVs increased substantially in all the inoculated anemones and no‐inoculum controls. *Labrenzia* increased in all inoculated anemones of all genotypes compared to the no‐inoculum controls, suggesting incorporation of these bacteria from both high and low FRS inocula. An increase of *Marinobacter* in the inoculated anemones also suggested incorporation from the inocula despite some moderate, albeit unsustained, increases in the control anemones. There were small but noticeable increases in *Micrococcus* in most inoculated anemones, particularly in the low FRS bacteria, but also increases in many uninoculated anemones. Increases in *Winogradskyella* in the inoculated anemones were clear but modest. Apart from *A. macleodii* and *Marinobacter*, none of the consortium members were maintained at higher than Day 0 levels for the duration of the experiment.

The bubble plots suggested there was little difference in the relative abundance of bacterial consortium members based on temperature. However, differences appeared to exist between the anemones based on inoculum, particularly in the relative abundance of *Labrenzia* immediately after Day 0. Paired GLM analyses confirmed that the bacterial communities of each anemone genotype were significantly different based on the inoculum they received (Table 3). This was short‐lived, with differences generally on Days 1 and 3 only. Therefore, when temperature ramping began on Day 8, there were no significant differences between the bacterial communities of the inoculated and uninoculated anemones.

**TABLE 3:** *p*‐values from GLM analyses comparing bacterial communities of anemones receiving different inocula (selected days only). Significant values are in bold; *α* = 0.05

Genotype	Comparison	Day 0	Day 1	Day 3	Day 8	Day 20	Day 31	Day 43
AIMS2	No‐inoculum control vs High FRS	0.058	**0.021**	**0.028**	0.078	0.253	0.286	0.255
	No‐inoculum control vs Low FRS	0.146	**0.027**	**0.020**	0.144	0.300	0.205	0.212
AIMS3	No‐inoculum control vs High FRS	0.086	**0.005**	**0.017**	**0.009**	0.329	0.172	0.484
	No‐inoculum control vs Low FRS	0.190	**0.046**	**0.027**	0.058	0.187	0.457	0.315
AIMS4	No‐inoculum control vs High FRS	0.124	**0.020**	**0.034**	0.082	0.175	0.628	0.320
	No‐inoculum control vs Low FRS	0.156	**0.006**	0.072	0.149	0.194	0.488	0.281

### Photosynthetic efficiency of Symbiodiniaceae

**FIGURE 4:**
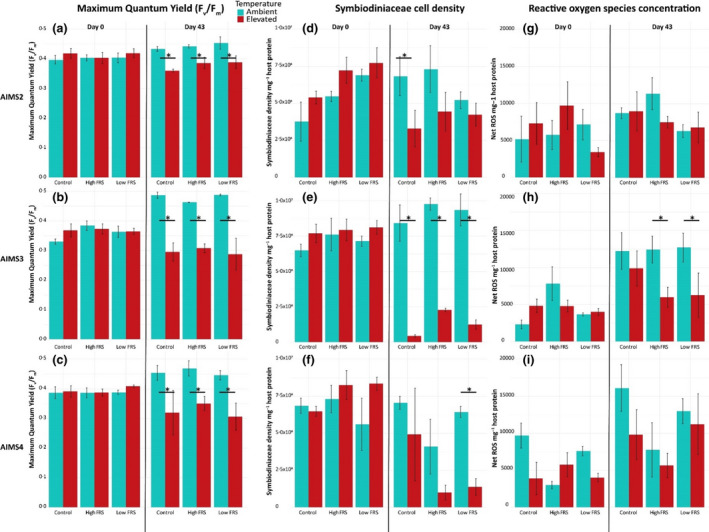
Photosynthetic efficiency (*F*_*v*_/*F*_*m*_) (a–c), Symbiodiniaceae density (d–f), and net ROS levels (g–i) for AIMS2 (a, d, g), AIMS3 (b, e, h) and AIMS4 (c, f, i) anemones under ambient (blue) and elevated (red) temperatures for day 0 (left side) and day 43 (right side) by inoculum. Asterisks indicate significant pairwise differences between ambient and elevated temperatures for the specified treatment with statistics reported in Table S4. There were no significant pairwise differences at day 0 or between inocula treatments on day 43. Error bars are standard error. For each bar, *n* = 3

Significant changes in *F*_*v*_ /*F*_*m*_ occurred in each anemone genotype according to the interaction of temperature and sampling day (Day 0 versus 43) (*F*_AIMS2(1,12)_ = 18.99, *p* = 0.0009; *F*_AIMS3(1,12)_ = 100.73, *p* < 0.0001; *F*_AIMS4(1,12)_ = 22.75, *p* = 0.0005), but not inoculation type. Pairwise comparisons showed that the significance of temperature varied by sampling day. At Day 43, *F*_*v*_/*F*_*m*_ was significantly lower for all anemones at elevated compared to ambient temperature for each inoculum, with no differences between inocula (Figure 4a–c**,** Table S3). There were no significant differences between temperature conditions on Day 0, which was expected as all anemones were at ambient temperature. By Day 12, *F*_*v*_/*F*_*m*_ reached levels approximating baseline values reported by Dungan et al. (2020), suggesting a full acclimation period of approximately 5 weeks (Figure S7). Thereafter, all anemones at ambient temperature maintained near‐normal *F*_*v*_/*F*_*m*_ levels.

### Symbiodiniaceae cell density

Symbiodiniaceae cell density changed significantly from Day 0 to Day 43 in each anemone genotype according to the interaction of sampling day and temperature (*F*_AIMS2(1,12)_ = 10.94, *p* = 0.0063; *F*_AIMS3(1,12)_ = 152.74, *p* < 0.0001; *F*_AIMS4(1,12)_ = 9.44, *p* = 0.0097), but not inoculation (Figure S8). To evaluate the interaction, and the ability of the high FRS inoculum to mitigate bleaching, we compared Symbiodiniaceae cell densities between inoculation treatments and temperature conditions for Day 0 and Day 43 by anemone genotype. Some significant differences in cell density were identified between Day 0 and Day 43 according to temperature for each genotype. There were no pairwise significant differences on Day 0 based on genotype (Figure 4d–f). On Day 43, Symbiodiniaceae density was significantly lower in AIMS3 anemones exposed to elevated temperature compared to the controls, regardless of inoculation (Figure 4e**,** Table S4), indicating that inoculation with high FRS bacteria did not mitigate bleaching. All AIMS2 (Figure 4d) and AIMS4 (Figure 4f) anemones had lower Symbiodiniaceae cell densities at elevated temperature compared to ambient temperature. However, the difference was significant only for the AIMS2 no‐inoculum control anemones and AIMS4 low FRS treated anemones (Table S4).

Since inoculation did not significantly influence Symbiodiniaceae cell density, the cell density data were pooled for each inoculum. This revealed that Symbiodiniaceae cell density was significantly lower for the anemones in the elevated temperature treatment at Day 43 compared to Day 0, indicating that all thermally stressed anemones bleached (Table S5). It also revealed that the AIMS3 anemones at ambient temperature underwent a significant increase in Symbiodiniaceae cell density from Day 0 to 43 (Table S5).

### ROS assay

ROS levels at Day 0 and Day 43 were significantly different according to the interaction of sampling day and temperature for AIMS3 (*F*_(1,12)_ = 8.16, *p* = 0.0144), or according to sampling day for AIMS4 (*F*_(1,12)_ = 12.82, *p* = 0.0038). There were no significant differences in net ROS across any variables for AIMS2. Nor were there any significant differences in the amount of ROS for the AIMS2 (Figure 4g) or AIMS4 (Figure 4i) anemones between ambient and elevated temperatures, regardless of inoculum, or between inoculation treatments in the elevated temperature condition on Day 0 or 43 (Table S4). Net ROS levels for AIMS3 anemones in the high and low FRS treatment at ambient temperature were significantly higher than those at elevated temperature, a trend we attribute to natural variability (Figure 4h).

### Symbiodiniaceae community characterization

Sequencing the Symbiodiniaceae ITS2 region produced 6.56 M reads across 108 *E*. *diaphana* samples (*n* = 54 for Day 0, *n* = 54 for Day 43, minimum 9086; mean 60,698, maximum 111,559 reads per sample) with no reads in the negative controls. After quality control, 2.35 M reads remained (minimum 213, mean 21,725, maximum 67,164 reads per sample). All anemones hosted *Breviolum minutum*, corresponding with previously reported GBR‐sourced *E*. *diaphana* (Dungan et al. 2020; Tortorelli et al. 2020). Four SymPortal ITS2 type profiles were identified: B1‐B1o‐B1p, B1‐B1a‐B1b, B1‐B1a‐B14a‐B1n, and B1‐B1a‐B1b‐B1g. All AIMS2 anemones exclusively hosted the B1‐B1o‐B1p ITS2 type profile Symbiodiniaceae, whereas 89% of AIMS3 anemones hosted B1‐B1a‐B1b, with 6% each of B1‐B1o‐B1p and B1‐B1a‐B1b‐B1g. AIMS4 anemones hosted either B1‐B1o‐B1p (50%), B1‐B1a‐B1b (44%), or B1‐B1a‐B14a‐B1n (6%). These profiles did not change during the experiment.

## DISCUSSION

Given the frequency of coral bleaching events, assisted evolution strategies, including the application of bacteria with putative beneficial properties, should be considered in addition to action plans to reduce carbon emissions, since without intervention coral reefs will not survive predicted climate change conditions. In our investigation of a microbial engineering strategy, we found that three applications of a high or low FRS bacterial consortium, tested in parallel with a no‐inoculum control, showed evidence of short‐term uptake into the *E*. *diaphana* microbiome. However, the incorporation of the consortium members was inconsistent, and none persisted in the anemone microbiome over time. Consequently, the failure of the high FRS bacteria to confer improved host thermal tolerance may have been due to their inability to integrate into the host microbiome for the full duration of the experiment. Importantly, there were no apparent physiological impacts (negative or positive) on the holobiont following inoculation, thus showing that the induced shifts in the abundance of native anemone microbiome members were not detrimental to holobiont health.

### Uptake of FRS bacteria by *E.* *diaphana* was uneven

Our dosing of 10^6^ bacterial cells ml^−1^ of each species in both the high and low FRS consortia is greater than reported bacterial carrying capacity of *E*. *diaphana*, which range from 10^3^–10^5^ bacteria anemone^−1^ (Costa et al. 2021; Dungan et al. 2021a). An increase in anemone‐associated *Labrenzia* provided the strongest evidence for the uptake of FRS bacterial by *E*. *diaphana*. *Labrenzia* are naturally abundant (~5%) in all the AIMS genotypes (Hartman et al. 2020; Dungan et al. 2021b) and are core members of the Symbiodiniaceae microbiome (Lawson et al. 2018). These bacteria may therefore have faced low inhibition from antagonistic interactions with resident bacteria (Rypien et al. 2010). Poor uptake of *Micrococcus* could have been caused by below‐target densities of those bacterial cultures in the inocula (Table 1), emphasizing the need to dose at consistently high cell densities, and possibly higher densities for some bacteria. *Alteromonas* are metabolically‐versatile copiotrophs (Pedler et al. 2014), rapidly responding to increases in dissolved organic matter and often dominating mesocosm experiments (McCarren et al. 2010). *A. macleodii* has been found to be highly abundant in *A. salina* feedstock used in our *E*. *diaphana* culture system (Hartman et al. 2020) and increased rapidly throughout the experiment, becoming dominant in inoculated and uninoculated anemones. This suggests that *A. macleodii* likely originated from the *A. salina* feedstock and multiplied due to the culture conditions.

### Uptake of FRS bacteria by *E.* *diaphana* was short‐lived

Post‐inoculation increases in the relative abundance of the FRS bacterial ASVs and significant differences between the bacterial communities of inoculated and uninoculated anemones on Days 1 and 3 suggested successful incorporation of the FRS bacteria by the anemones. However, this was short‐lived. The failure of bacterial uptake by the host may be due to failure of the selected bacteria to be recognized as symbionts by the host, control of bacterial adhesion as described by the bacteriophage adherence to mucus model (Barr et al. 2013), or founder effects whereby existing bacterial communities influence the identity of new associates (Apprill et al. 2012). Further, the ciliated surface of *E*. *diaphana* may play a role in preventing bacterial adhesion (Costa et al. 2021). The three‐dose strategy employed in the present study was similar to previous cnidarian experiments. For example, Rosado et al. (2018) inoculated coral samples with a bacterial cocktail twice, 5 days apart, Damjanovic et al. (2019b) inoculated coral larvae seven times, at 3–4 day intervals, and Doering et al. (2021) completed microbiome transplants three times over 3 days for *Porites* coral fragments. However, these studies did not assess the uptake of inoculated bacteria between doses. Although previous studies have demonstrated that bacterial inoculation can protect *E*. *diaphana* from pathogenic infection (Alagely et al. 2011; Zaragoza et al. 2014), incorporation of the selected bacteria into the host microbiome was not assessed. Recently, Costa et al. (2021) showed that *E*. *diaphana* is resistant to microbiome changes as transplantation of either the *Acropora humilis* or *Porites* sp. associated microbiomes failed to shift the community composition compared to *Exaiptasia* microbiome controls.

For introduced bacteria to be incorporated into the host microbiome, they must be attracted by the host’s chemical cues, be recognized by the host, and be resistant to any antimicrobial compounds present (Krediet et al. 2013). Since our FRS bacteria were host‐derived, it is possible that they met these criteria, but equally feasible that these individuals were transiently associated. Regardless, introducing the FRS bacteria and creating a stable shift in the host’s microbial community proved challenging. In the few studies that have investigated the onset of bacterial symbiosis with coral hosts, the focus has been on changes in bacterial community structure over time (Apprill et al. 2009; Damjanovic et al. 2019a), and there is evidence that some bacterial symbionts are taken up more readily than others (Apprill et al. 2012). Future studies that examine the chemical cues produced by coral hosts or bacteria and the chemicals involved in bacterial recognition will improve our understanding of the biochemical requirements for bacterial uptake, and hence our ability to induce stable changes (Kvennefors et al. 2008).

### Thermal stress, ROS and bleaching

Thermally induced bleaching occurred for all genotypes as there was a significant reduction of Symbiodiniaceae cell density and significant decline in photosynthetic efficiency on experimental Day 43 for anemones in the elevated temperature condition compared to ambient. Significant reductions in *F*_*v*_/*F*_*m*_ and Symbiodiniaceae cell density for all anemones at elevated temperature occurred regardless of inoculated or uninoculated treatment. While the ROS data suggest some neutralization of ROS, the overall variation in the ROS values advises caution not to overinterpret these results.

The bleaching response of *E*. *diaphana* is thought to be initiated by an increased production of ROS by their algal endosymbionts during periods of stress (Lesser 1997; Weis 2008). Contrary to the ‘oxidative stress theory of bleaching’ (Downs et al. 2002), elevated temperature did not lead to an increase in net ROS (Figure 4g–i). Instead, bleaching of the AIMS3 anemones at elevated temperature in the high and low FRS treatments was accompanied by significant declines in net ROS. This observation corresponds with other studies that have shown bleaching can occur without photosynthetically produced ROS (Tolleter et al. 2013) and with discrepancies in enzymatic antioxidant activity between host and symbiont tissue portions (Krueger et al. 2015). Our data support the suggestion that bleaching does not require an influx of ROS from the algal symbiont to the host (Nielsen et al. 2018), and raises questions about the importance of symbiont‐derived ROS in initiating bleaching in *E*. *diaphana*. Given the novel finding that elevated ROS levels were not associated with bleaching in *E*. *diaphana*, this organism may not be an ideal candidate for testing an FRS bacterial consortium to mitigate climate change in cnidarians.

### Bleaching susceptibility differed by genotype

As the introduced bacteria were not retained by the anemones, no correlation can be drawn between differences in bleaching tolerance and inoculation. However, the variation in bleaching intensity between genotypes is noteworthy as AIMS2 lost fewer Symbiodiniaceae under elevated temperature compared to AIMS3 and AIMS4. It is possible that differences in bleaching tolerance were driven by differences in the *in hospite* Symbiodiniaceae communities harboured by each anemone genotype (Howells et al. 2012; Hawkins et al. 2016). The AIMS2 anemones, which did not bleach, harboured a distinct Symbiodiniaceae ITS2 type profile, B1‐B1o‐B1p. Very few AIMS3 individuals had this ITS2 type profile and bleached heavily, whereas 50% of the AIMS4 anemones had this ITS2 type profile and displayed high variation in their bleaching response. These data suggest that Symbiodiniaceae with the B1‐B1o‐B1p ITS2 type profile could confer some thermal resilience to the holobiont. Alternatively, the anemone genotypes may have different bleaching susceptibilities independent of their Symbiodiniaceae type profile (Gabay et al. 2019). Previous work on corals (Quigley et al. 2018) and GBR‐sourced *E*. *diaphana* (Tortorelli et al. 2020) has shown that the mechanism of recognition and incorporation of Symbiodiniaceae into the holobiont is influenced by both algal symbiont and host. Observations from the present study indicate that genotype and algal symbiont type will be critical in future experiments seeking to induce or mitigate bleaching in *E. diaphana*.

### Limitations of the study, and recommendations for future microbial engineering work

Although there was evidence for increased relative abundance of some consortium members in the host microbiome, the key limitation of the present study was the inability of the bacteria to persist after inoculation. Bacteria delivery via bioencapsulation in *A. salina* is used in aquaculture to ensure dosed bacteria are ingested (Hai et al. 2010), and a method for corals using rotifers has been reported (Assis et al. 2020). Researchers using probiotics to treat stony coral tissue loss disease (SCTLD) in the field have also tested strategies such as weighted enclosures, paste applied directly to SCTLD lesions, and slow release beads to improve bacteria‐coral contact and maintain probiotic concentrations in the open marine environment (Smithsonian Marine Station 2020). Microencapsulation of beneficial bacteria in alginate has been explored in the aquaculture industry, with high efficiency (80% of bacteria survived alginate encapsulation), retention (40% of encapsulated bacteria survived storage at 22°C for 30 days), and storage survival (over 90% survival of bacteria after 1 month storage at 4°C) (Rosas‐Ledesma et al. 2012). These approaches may address the issues of dilution and inadequate uptake of putative beneficial bacteria by coral and warrant further investigation. Furthermore, of the nearly 1000 bacteria isolated from *E*. *diaphana* (Dungan et al. 2021a) and the thousands of bacterial cells they can carry (Costa et al. 2021), only 12 were used in this study. More work is needed to explore the potential beneficial roles of the other bacteria, which may be possible with advances in metagenomic sequencing and the assembly of bacterial metagenome‐assembled genomes.

Because closely related bacterial strains can have an identical sequence in the short 16S rRNA gene region examined with metabarcoding, such as the high and low FRS conspecific pairs used in this study, future studies could be enhanced by quantifying consortium members using qPCR with strain specific primers to determine whether each member was retained in the host microbiome after inoculation. An element of the present study that we recommend as good practice is the use of a negative inoculum (here, low FRS). Inclusion of a negative inoculum allowed us to account for differences in anemone response to thermal stress due to the introduction of an additional source of nutrition as heterotrophy can reduce the impact of thermal stress on corals (Grottoli et al. 2006; Aichelman et al. 2016).

To date, few studies have investigated the ability of bacteria to increase cnidarian thermal tolerance. For this type of assisted evolution strategy to be feasible, interventions must minimize risk (NASEM 2019). One of the unknown risks in microbial engineering is its impact on holobiont physiology as the introduction of high numbers of microbes to the holobiont could trigger unintended consequences. Critically, our results suggest that inoculation with a consortium of host‐derived bacteria does not negatively impact anemone or Symbiodiniaceae physiology. Some members of our inocula remained with the host after dosing, but only for a short period of time. Therefore, while inoculation with FRS bacteria did not mitigate bleaching in heat‐exposed anemones, this cannot be attributed to an inability of the FRS bacteria to confer improved thermal tolerance, which remains unproven. Future studies that maintain elevated levels of introduced bacteria with appropriate controls to consider heterotrophy will provide clearer insights into the potential of the coral bleaching mitigation strategy proposed here.

## CONFLICT OF INTEREST

The authors declare no conflicts of interest.

## Supporting information


Supplementary File S1
Table S1–S5
Figure S1–S10

## Data Availability

Raw Illumina MiSeq data are available under NCBI BioProject ID PRJNA630329 (https://www.ncbi.nlm.nih.gov/bioproject/PRJNA630329).
