# Supplementary material for: Exploring microbiome engineering as a strategy for improved thermal tolerance in Exaiptasia diaphana
Source: J Appl Microbiol. 2022 Feb 19;132(4):2940–56. doi: 10.1111/jam.15465 (PMC9303619; doi:10.1111/jam.15465)
Supplement: Supplementary file 1 — Supplementary File S1 Table S1–S5 Figure S1–S10 [file JAM-132-2940-s001.docx]

**Supplementary File S1**

Isolation and identification of candidate bacteria

Great Barrier Reef-origin *Exaiptasia diaphana* were taken from The University of Melbourne anemone culture collection (Dungan et al. 2020b) as source material for the selected bacteria. Sixteen replicates from each of four *E. diaphana* genotypes (AIMS1-4) were collected using sterile pipettes and transferred to sterile 12-well plates (CLS3513, Corning, USA) filled with 0.2 µm-filtered Red Sea Salt™ (RSS) seawater reconstituted with reverse osmosis water at ~34 parts per thousand (ppt) salinity, hereafter ‘fRSS’. The anemones were left for 30 min to remove loosely associated bacteria, then transferred into sterile glass Dounce homogenizers filled with 1 ml of fRSS and ground into a slurry. Serial dilutions were created from each homogenate at 10^-1^, 10^-2^, 10^-3^ and 10^-4^. From each dilution, 50 µl was spread onto three marine agar (MA) plates (PP2315, Thermo Fisher, Australia) and three R2A agar plates (CM0906, Thermo Fisher, Australia) made with 40 g L^-1^ RSS to 34 ppt salinity. All plates were incubated at the anemone rearing temperature, 26°C. After one-week of incubation, cells were picked from individual colonies on plates with <100 colony forming units, and sub-cultured to purification on media consistent with their original growth. A colony from each pure culture isolate was suspended in 40% glycerol and aliquoted into 1.2 ml cryotubes for storage at –80°C.

Each bacterial culture was taxonomically identified by Sanger sequencing of the 16S rRNA genes. DNA for sequencing was prepared as follows: cells from each pure culture were suspended in 20 µl sterile H_2_O and denatured at 95°C for 10 min. Each suspension was then centrifuged at 2,000 × *g* at 4°C for 2 min. PCRs were set up using 2 µl of the resulting supernatant as template DNA in 40 µl reactions also containing 0.5 U Mango Mix™ (Bioline, Australia), and 0.25 µmol l^-1^ of universal bacterial primers 27F (5’ AGAGTTTGATCMTGGCTCAG 3’) and 1492R (5’ TACGGYTACCTTGTTACGACTT 3’) (Lane 1991). PCR thermal cycler settings were: 1 × 95°C for 5 min; 35 cycles of 95°C for 1 min, 50°C for 1 min, and 72°C for 1 min; 1 × 72°C for 10 min. The PCR products were then purified and sequenced on an ABI sequencing instrument at Macrogen Inc. (Seoul, South Korea) using the 1492R primer. The sequence data for each isolate was trimmed and aligned against sequences in the NCBI GenBank database with BLAST (Altschul et al. 1990) to establish taxonomic identity.

Candidate selection by qualitative free radical scavenging (FRS) assay

To qualitatively assess isolate free radical scavenging (FRS) ability, each isolate was grown on solid MA or R2A media for 48 hr at 26°C, then incubated overnight with a sterile Whatman #1 filter in contact with the bacterial colonies. The filters were removed with forceps and allowed to dry for 30 min, then wet with 500 µl of a 0.2 mmol l^-1^ DPPH-methanol (D9132, Sigma-Aldrich, Australia) solution. DPPH is a stable free radical that it is purple in its oxidized state but becomes white-yellow when reduced by antioxidants and has been used to identify antioxidant marine bacteria (Takao et al. 1994; Velho-Pereira et al. 2015). Several drops of 0.1% (w/v) L-ascorbic acid (A7631, Sigma-Aldrich, Australia) were also applied to a filter paper and tested as a positive control. The response of each isolate to the DPPH solution was recorded for 3 min after application. Appearance of a white-yellow halo around individual colonies within 1 min was judged a positive response (**Fig. S10a**). Appearance of a halo after 1-3 min was judged a weak positive response. Failure to form a halo was judged a negative response (**Fig. S10b**).

Candidate selection by quantitative free radical scavenging (FRS) assay

To quantitatively assess isolate FRS ability, each was grown on sold media, as described above. From each isolate, cells were picked from single colonies and inoculated into triplicate 250 ml conical flasks containing 50 ml sterile R2A broth supplemented with RSS to 34 ppt (**Table S5**). The FRS phenotype of each high and low FRS bacteria was similar when incubated at 26°C or 37°C (**Table S1**); since the bacteria grew more rapidly at 37°C, cultures were incubated at 37°C for 48 h at 150 rpm in an orbital incubator (OM11, Ratek, Australia). Uninoculated broth was simultaneously incubated to confirm the absence of media contamination. After 48 hrs, OD600 measurements were collected from each culture and media blank (CLARIOstar PLUS plate-reader, BMG Labtech, Australia). The cultures were then centrifuged at 3000 × *g* at 4°C for 30 min to pellet the bacterial cells. The cell free supernatant (CFS) was collected, frozen at –80˚C, and freeze-dried (Alpha 1-4 LDplus, Martin Christ, Germany). The freeze-dried CFS was kept under inert gas in darkness until analysis. Antioxidants from the CFS were extracted by suspending in 100% methanol to a concentration of 50 mg ml^-1^, sonicating for 5 min, and then centrifuging at 3000 x *g* for 5 min at 4˚C. Quantitative DPPH assays were then performed on each CFS extract mixed with 0.2 mmol l^-1^ DPPH-methanol in a 1:1 ratio to a volume of 1 ml. Each sample was vortexed and incubated for 30 min at room temperature, then vortexed again briefly. Three 300 µl replicates of each sample were transferred to a 96 well plate, and absorbance was measured at 517 nm. Percentage FRS was calculated according to the following formula, where control = no-sample 0.2 mmol l^-1^ DPPH-methanol:

% FRS activity = (control – sample) / control × 100

Reaction blanks of 100% methanol, and positive controls of 0.01%–0.001% (w/v) L-ascorbic acid were included in each plate. High FRS strains were defined as those with a significantly greater % FRS activity compared to a media negative control and/or a congeneric/conspecific low FRS strain. All low FRS strains had % FRS values that were not significantly different from the media negative controls.

**TABLE S1** R2A broth adjusted for growth of marine bacteria. Final pH = 7.2 +/- 0.2 at 25°C. Final salinity = ~34 ppt.

| **Component** | **grams L^–1^** | **Supplier** |
| --- | --- | --- |
| Casein acid hydrolysate | 0.500 | C0501, Sigma Aldrich, Australia |
| Yeast extract | 0.500 | LP0021, Oxoid, Thermo Fisher, Australia |
| Proteose peptone | 0.500 | 211684, Thermo Fisher, Australia |
| Dextrose | 0.500 | G360, Austratec, Australia |
| Starch, soluble | 0.500 | AJA526, Univar, Australia |
| Dipotassium phosphate | 0.300 | P3786, Sigma Aldrich, Australia |
| Magnesium sulfate | 0.024 | M2643, Sigma Aldrich, Australia |
| Sodium pyruvate | 0.300 | P2256, Sigma Aldrich, Australia |
| Red Sea Salt™ | 40.00 | R11065, Red Sea, USA |

**TABLE S2** Free radical scavenging (FRS) abilities for each selected strain at 37°C (after 2 days) and 26°C (after 7 days) measured as the percent reduction in absorbance at 517 nm compared to the DPPH control. R2A broth control data is included. Because cultures were required more frequently than seven days and the FRS ability was similar at both temperatures, we opted to grow the bacteria at 37°C to build biomass for inoculations.

| Strain | Genus | FRS at 37°C (% ± SE) | n at 37°C | FRS at 26°C (% ± SE) | n at 26°C |
| --- | --- | --- | --- | --- | --- |
| MMSF01163 | *Alteromonas* | 61.7 ± 5.2 | 3 | 66.7 ± 5.6 | 3 |
| MMSF00404 | *Alteromonas* | 35.5 ± 13.7 | 4 | 31.0 ± 7.7 | 3 |
| MMSF00958 | *Alteromonas* | 62.0 ± 4.2 | 3 | 70.0 ± 9.7 | 3 |
| MMSF00257 | *Alteromonas* | 30.3 ± 0.9 | 3 | 38.5 ± 4.6 | 3 |
| MMSF00132 | *Labrenzia* | 53.6 ± 8.7 | 8 | 79.7 ± 6.0 | 3 |
| MMSF00249 | *Labrenzia* | 14.0 ± 3.6 | 3 | 8.3 ± 2.9 | 3 |
| MMSF01190 | *Marinobacter* | 62.0 ± 4.5 | 3 | 64.7 ± 5.8 | 3 |
| MMSF00964 | *Marinobacter* | 43.7 ± 5.8 | 3 | 22.4 ± 4.8 | 3 |
| MMSF00068 | *Micrococcus* | 56.3 ± 7.3 | 6 | 73.2 ± 9.4 | 3 |
| MMSF00107 | *Micrococcus* | 38.0 ± 8.0 | 3 | 22.2 ± 6.5 | 3 |
| MMSF00046 | *Winogradskyella* | 73.3 ± 1.9 | 3 | 69.5 ± 10.3 | 3 |
| MMSF00910 | *Winogradskyella* | 36.0 ± 3.5 | 3 | 18.6 ± 4.9 | 3 |
| R2A negative | NA | 27.2 ± 2.3 | 12 | 37.1 ± 7.2 | 6 |

**TABLE S3** The 20 most abundant contaminant ASVs identified by decontam across all anemone samples. Some genera have members associated with human skin, suggesting the origin of those putative contaminants e.g. *Cutibacterium*.

|  | Phylum | Class | Order | Family | Genus | Relative abundance (%) |
| --- | --- | --- | --- | --- | --- | --- |
| 1 | Proteobacteria | Gammaproteobacteria | Betaproteobacteriales | Burkholderiaceae | *Pelomonas* | 0.44 |
| 2 | Proteobacteria | Alphaproteobacteria | Sphingomonadales | Sphingomonadaceae | *Sphingomonas* | 0.15 |
| 3 | Proteobacteria | Gammaproteobacteria | Pseudomonadales | Moraxellaceae | *Acinetobacter* | 0.13 |
| 4 | Proteobacteria | Gammaproteobacteria | Xanthomonadales | Xanthomonadaceae | *Stenotrophomonas* | 0.08 |
| 5 | Proteobacteria | Gammaproteobacteria | Pseudomonadales | Pseudomonadaceae | *Pseudomonas* | 0.06 |
| 6 | Actinobacteria | Actinobacteria | Propionibacteriales | Propionibacteriaceae | *Cutibacterium* | 0.03 |
| 7 | Firmicutes | Clostridia | Clostridiales | Ruminococcaceae | *Ruminococcaceae* | 0.03 |
| 8 | Proteobacteria | Gammaproteobacteria | Pseudomonadales | Moraxellaceae | *Enhydrobacter* | 0.03 |
| 9 | Firmicutes | Bacilli | Lactobacillales | Streptococcaceae | *Streptococcus* | 0.02 |
| 10 | Proteobacteria | Gammaproteobacteria | Betaproteobacteriales | Burkholderiaceae | *Ralstonia* | 0.02 |
| 11 | Proteobacteria | Alphaproteobacteria | Sphingomonadales | Sphingomonadaceae | *Sphingomonas* | 0.02 |
| 12 | Proteobacteria | Gammaproteobacteria | Pseudomonadales | Moraxellaceae | *Acinetobacter* | 0.02 |
| 13 | Actinobacteria | Actinobacteria | Propionibacteriales | Propionibacteriaceae | *Cutibacterium* | 0.02 |
| 14 | Firmicutes | Clostridia | Clostridiales | Family XI | *Finegoldia* | 0.01 |
| 15 | Proteobacteria | Gammaproteobacteria | Betaproteobacteriales | Burkholderiaceae | *Cupriavidus* | 0.01 |
| 16 | Bacteroidetes | Bacteroidia | Flavobacteriales | Weeksellaceae | *Chryseobacterium* | 0.01 |
| 17 | Actinobacteria | Actinobacteria | Corynebacteriales | Corynebacteriaceae | *Corynebacterium* | 0.01 |
| 18 | Actinobacteria | Actinobacteria | Propionibacteriales | Propionibacteriaceae | *Cutibacterium* | 0.01 |
| 19 | Firmicutes | Bacilli | Bacillales | Staphylococcaceae | *Staphylococcus* | 0.01 |
| 20 | Firmicutes | Bacilli | Bacillales | Bacillaceae | *Bacillus* | 0.01 |
| 21-209 | Other |  |  |  |  | 0.24 |
|  |  |  |  |  | Total | **1.36** |

**TABLE S4** Pairwise comparisons for photosynthetic efficiency, Symbiodiniaceae cell density and net ROS for Day 43. Only significant values (α = 0.05) are shown.

| **Genotype** | **Parameter** | **Inoculum** | **Temperature** | **Day** | **df** | **t-ratio** | **p value** |
| --- | --- | --- | --- | --- | --- | --- | --- |
| AIMS2 | Photosynthetic Efficiency | Low FRS | Ambient>Elevated | 43 | 12 | 3.016 | 0.0107 |
| AIMS2 | Photosynthetic Efficiency | Control | Ambient>Elevated | 43 | 12 | 3.391 | 0.0054 |
| AIMS2 | Photosynthetic Efficiency | High FRS | Ambient>Elevated | 43 | 12 | 2.61 | 0.0228 |
| AIMS2 | Symbiodiniaceae Cell Density | Control | Ambient>Elevated | 43 | 12 | 2.469 | 0.0296 |
| AIMS3 | Photosynthetic Efficiency | Low FRS | Ambient>Elevated | 43 | 12 | 6.456 | <0.0001 |
| AIMS3 | Photosynthetic Efficiency | Control | Ambient>Elevated | 43 | 12 | 4.995 | 0.0003 |
| AIMS3 | Photosynthetic Efficiency | High FRS | Ambient>Elevated | 43 | 12 | 6.188 | <0.0001 |
| AIMS3 | Symbiodiniaceae Cell Density | Low FRS | Ambient>Elevated | 43 | 12 | 8.009 | <0.0001 |
| AIMS3 | Symbiodiniaceae Cell Density | Control | Ambient>Elevated | 43 | 12 | 7.399 | <0.0001 |
| AIMS3 | Symbiodiniaceae Cell Density | High FRS | Ambient>Elevated | 43 | 12 | 7.891 | <0.0001 |
| AIMS3 | net ROS | Low FRS | Ambient>Elevated | 43 | 12 | 2.603 | 0.0231 |
| AIMS3 | net ROS | High FRS | Ambient>Elevated | 43 | 12 | 2.594 | 0.0235 |
| AIMS4 | Photosynthetic Efficiency | Low FRS | Ambient>Elevated | 43 | 12 | 3.269 | 0.0067 |
| AIMS4 | Photosynthetic Efficiency | Control | Ambient>Elevated | 43 | 12 | 2.755 | 0.0174 |
| AIMS4 | Photosynthetic Efficiency | High FRS | Ambient>Elevated | 43 | 12 | 3.317 | 0.0086 |
| AIMS4 | Symbiodiniaceae Cell Density | Low FRS | Ambient>Elevated | 43 | 12 | 2.802 | 0.016 |

**TABLE S5** Symbiodiniaceae cell density comparisons between Days 0 and 43 for AIMS2-4 anemones by temperature treatment. All inoculum treatments were pooled. Significant values are in bold; α = 0.05.

| Genotype | Data: Contrast | Treatment | df | t.ratio | p.value |
| --- | --- | --- | --- | --- | --- |
| AIMS2 | Ambient: Day 0-Day 43 | All | 12 | 1.29 | 0.2187 |
| AIMS2 | Elevated: Day 0-Day 43 | All | 12 | 3.379 | **0.0055** |
| AIMS3 | Ambient: Day 0-Day 43 | All | 12 | 4.224 | **0.0012** |
| AIMS3 | Elevated: Day 0-Day 43 | All | 12 | 13.255 | **<0.0001** |
| AIMS4 | Ambient: Day 0-Day 43 | All | 12 | 0.700 | 0.4970 |
| AIMS4 | Elevated: Day 0-Day 43 | All | 12 | 5.045 | **0.0003** |

**FIG S1** Water temperature daily averages (2 m depth) from St Crispin Reef (AIMS 2019). The graph is illustrative for summer heatwave conditions and shows an increase in SST for a northern part of the GBR in summer 2016; a year that saw “unprecedented” mass coral bleaching (Hughes et al. 2017b).

**FIG S2** Relative abundance of order-level bacterial taxa in the AIMS2 anemones. The 20 most abundant orders are shown, with all other orders shown as Other. For each bar, *n* = 3.

**FIG S3** Relative abundance of order-level bacterial taxa in the AIMS3 anemones. The 20 most abundant orders are shown, with all other orders shown as Other. For each bar, *n* = 3.

**FIG S4** Relative abundance of order-level bacterial taxa in the AIMS4 anemones. The 20 most abundant orders are shown, with all other orders shown as Other. For each bar, *n* = 3.

**FIG S5** Relative abundance of the inoculated bacteria in the AIMS3 anemones, identified to the ASV level. Blue genus names = data for ambient temperature anemones (26°C). Pink genus names = data for elevated temperature anemones (26°C–31.5°C). Inoculations were performed at Days 0, 2, and 7 and are indicated by stars. For each bubble, *n* = 3.

**FIG S6** Relative abundance of inoculated bacteria in the AIMS4 anemones, identified to the ASV level. Blue genus names = data for ambient temperature anemones (26°C). Pink genus names = data for elevated temperature anemones (26°C–31.5°C). Inoculations were performed at Days 0, 2, and 7 and are indicated by stars. For each bubble, n = 3.

**FIG S7** F_v_/F_m_ at ambient (26°C) and elevated (26°C–31.5°C) temperature for all experimental treatments for the three genotypes. Inoculations were performed on Days 0, 2, and 7. For each datapoint, *n*= 3. Error bars ± 1 SEM.

**FIG S8** Symbiodiniaceae cell density at ambient (26°C) and elevated (26°C–31.5°C) temperature for all experimental treatments and all three genotypes. Inoculations were performed at Days 0, 2, and 7. For each datapoint, *n* = 3. Error bars ± 1 SEM.

**FIG S9** ROS levels at ambient (26°C) and elevated (26°C–31.5°C) temperature for all treatments within each genotype. Inoculations were performed at Days 0, 2 and 7. Due to sampling error, Day 40 ROS measurements were not completed and were excluded from the data set. For each datapoint, *n*= 3. Error bars ± 1 SEM.

(a)
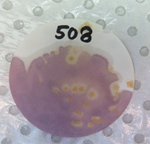
 (b)
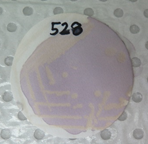


**FIG S10** Examples of DPPH qualitative assay results: (a) Positive indication of FRS ability for a bacterial isolate due to appearance of halo in <1 min; (b) Negative indication of FRS ability for a bacterial isolate due to absence of halo after >3 min.
